# Bacterial Endophytes from Legumes Native to Arid Environments Are Promising Tools to Improve *Mesorhizobium*–Chickpea Symbiosis under Salinity

**DOI:** 10.3390/biology13020096

**Published:** 2024-02-03

**Authors:** Roukaya Ben Gaied, Imed Sbissi, Mohamed Tarhouni, Clarisse Brígido

**Affiliations:** 1 Laboratory of Pastoral Ecosystems and Promotion of Spontaneous Plants and Associated Micro-Organisms, Institute of Arid Lands, University of Gabes, Medenine 4119, Tunisia; roukaya.bengaied@gmail.com (R.B.G.); sbissimed@gmail.com (I.S.); medhtarhouni@yahoo.fr (M.T.); 2 MED-Mediterranean Institute for Agriculture, Environment and Development, Universidade de Évora, Pólo da Mitra, Ap. 94, 7006-554 Évora, Portugal; 3 MED-Mediterranean Institute for Agriculture, Environment and Development & CHANGE-Global Change and Sustainability Institute, Institute for Advanced Studies and Research, Universidade de Évora, Pólo da Mitra, Ap. 94, 7006-554 Évora, Portugal

**Keywords:** abiotic stress, root exudates, salt-stress, endophytic bacteria, grain legume, inoculants

## Abstract

**Simple Summary:**

Soil salinity is increasing worldwide and is a major environmental issue that affects soil fertility and agricultural productivity. In this study, we show that the early events of the *Mesorhizobium*-chickpea symbiosis are negatively affected by salinity due to substantial changes in the composition of phenolic compounds of chickpea root exudates, which in turn affect the perception and response of its microsymbiont. In addition, the use of non-rhizobial nodule endophytes from legumes native to arid regions proved to be a promising strategy to improve legume growth and to enhance *Mesorhizobium*-chickpea symbiosis under salinity. In conclusion, this study helps to extend our knowledge on the detrimental effects of salinity on legume-rhizobium symbiosis and highlights the potential use of beneficial nodule bacteria as biological tools to maintain a healthier rhizobium–legume symbiosis, thus enhancing the growth of salt-sensitive legumes under salinity conditions.

**Abstract:**

Symbiotic nitrogen fixation is a major contributor of N in agricultural ecosystems, but the establishment of legume–rhizobium symbiosis is highly affected by soil salinity. Our interest is focused on the use of non-rhizobial endophytes to assist the symbiosis between chickpea and its microsymbiont under salinity to avoid loss of production and fertility. Our aims were (1) to investigate the impact of salinity on both symbiotic partners; including on early events of the *Mesorhizobium*-chickpea symbiosis, and (2) to evaluate the potential of four non-rhizobial endophytes isolated from legumes native to arid regions (*Phyllobacterium salinisoli*, *P. ifriqiyense*, *Xanthomonas translucens,* and *Cupriavidus respiraculi*) to promote chickpea growth and nodulation under salinity. Our results show a significant reduction in chickpea seed germination rate and in the microsymbiont *Mesorhizobium ciceri* LMS-1 growth under different levels of salinity. The composition of phenolic compounds in chickpea root exudates significantly changed when the plants were subjected to salinity, which in turn affected the *nod* genes expression in LMS-1. Furthermore, the LMS-1 response to root exudate stimuli was suppressed by the presence of salinity (250 mM NaCl). On the contrary, a significant upregulation of *exoY* and *otsA* genes, which are involved in exopolysaccharide and trehalose biosynthesis, respectively, was registered in salt-stressed LMS-1 cells. In addition, chickpea co-inoculation with LMS-1 along with the consortium containing two non-rhizobial bacterial endophytes, *P. salinisoli* and *X. translucens*, resulted in significant improvement of the chickpea growth and the symbiotic performance *of* LMS-1 under salinity. These results indicate that this non-rhizobial endophytic consortium may be an appropriate ecological and safe tool to improve chickpea growth and its adaptation to salt-degraded soils.

## 1. Introduction

Soil salinity is increasing worldwide and is a global issue threatening land productivity and food security [1]. According to data from FAO, more than 833 million hectares of soil worldwide are now considered affected by salt, and estimates indicate that more than 10% of agricultural land is affected by salt [2]. Therefore, it is crucial to better understand the deleterious effect of soil salinity on the various plant processes, as well as to develop strategies that allow mitigating the negative effects on the production and sustainability of crops in these salt-affected soils.

Most legumes are highly sensitive to salinity, as are the processes of nodulation and nitrogen fixation by legume microsymbionts. For legumes, the maintenance of a healthy and balanced symbiosis with rhizobia is a crucial factor to survive stress damage [3,4]. This symbiotic interaction presents a biological source of nitrogen (N), where atmospheric N is converted to ammonium inside the root nodules [5]. The nodulation process involves a series of signaling cascade, starting with the release of root exudates (RE) from the plant roots into the rhizosphere [6,7]. The perception of these chemical signals by rhizobia leads to the expression of multiple sets of genes designated as *nod* genes [8]. The *nodD* gene encodes for the transcriptional factor regulating the expression of the rest of *nod* box genes in response to specific compounds from the RE [9]. Once upregulated, *nodD* will activate the expression of *nodABC* genes encoding for proteins involved in the biosynthesis of Nod factors (lipo-chitooligosaccharides), where *nodA* and *nodC* are responsible for the synthesis of the backbone structure of these Nod factors (NF) [7,10]. The recognition of the specific structure of NF by the host’s receptor kinases induces different symbiotic events, including root hair curling and the development of infection threads, which represent the entry point of rhizobia towards the root cortex, thus, the induction of nodule primordia and the differentiation of bacteroids responsible for nitrogen fixation under optimal growth conditions [11].

In saline soils, a decreased nodulation and nitrogen supply have been registered in grain legumes [12,13,14]. This lack of symbiotic efficiency can be attributed to a disruption in the molecular signaling between the two partners or a failure in the infection process because of salt stress [15,16]. Plants exposed to ionic stress undergo various metabolic changes leading to an alteration of the composition of their RE. With such chemical modifications, the plant risks losing connection with its microsymbiont. In soybean plants for example, the pre-activation of *Bradyrhizobium japonicum* with genistein, a flavonoid compound previously reported as *nod* gene inducer, improved the nodulation and nitrogen content of plants grown under salinity [17]. These findings suggest that plants growing under stress had a low ability to produce this inducer, which resulted in the inhibition of symbiotic infection. In another report, a decrease in the induction level of *nod* genes was observed in rhizobia exposed to bean RE collected under salt stress compared to those collected under normal conditions [18]. In addition, several studies demonstrated the negative effects of salinity on root infection and colonization by rhizobia. In a study conducted by Zahran and Sprent [19], sodium chloride caused an inhibition of root hairs expansion and curling due to plasmolysis, which resulted in a decreased nodule number by about 50%. Moreover, the establishment of nodules in legumes grown under salt stress does not guarantee a normal nitrogen fixation process. Contrarily, several studies demonstrated the inhibition of nitrogenase activity under salinity. Serraj [20] associated the inhibition of nitrogenase activity by NaCl with the decrease of nodule permeability to oxygen. Similarly, a decline in nitrogenase activity was also reported in chickpea plants growing under 50 mM of NaCl [21]. 

To break the eco-physiological barriers of salt stress in crop cultivation, it is necessary to improve the tolerance of legume–rhizobium symbiosis to salinity. Considering the notable susceptibility of rhizobia single inoculants to NaCl, numerous studies have been focusing on the identification and application of new salt-tolerant bacterial inoculum with various plant growth promoting (PGP) features [22,23,24]. Qualified as plant growth promoting bacteria (PGPB), these isolates belong to various species and come from different origins, mainly the plant microbiome. PGPBs exert their influence on plant growth promotion through various direct and indirect mechanisms, including nitrogen fixation, siderophores and phytohormones production, phosphate solubilization, and induction of plant immune system against phytopathogens [25,26]. The co-inoculation of grain legumes with rhizobia and other PGPBs has been found to elicit diverse positive effects, particularly in plants growing under abiotic stress [24,27].

Previous studies reported multiple aspects of salinity stress alleviation in crops after treatment with PGPBs such as *Pseudomonas*, *Enterobacter*, *Bacillus*, *Burkholderia*, *Azospirillum*, among others [28,29,30,31,32]. However, there is still a lack of knowledge concerning the role of endophytes inhabiting rhizobia-nodules in the establishment of legume–rhizobium symbiosis and whether their presence or absence could interfere with the nodulation and nitrogen fixation process, in particularly under stress conditions. Bacterial endophytes from root nodules of wild legumes, grown in arid soils, have not been thoroughly explored for their potential in the field of plant–bacteria interactions. This unique microbial community has evolved to withstand the extreme environmental conditions of arid regions, including high temperatures and salinity conditions [33,34]. With such an inherent adaptability to various stressors, these endophytes may represent a valuable source of biofertilizers for legumes cultivation in saline soils. 

Within this context, the present study aimed to investigate the impacts of salt stress on the early symbiotic interaction between rhizobium and its host, as well as to determine the capacity of bacterial endophytes derived from root nodules of wild legumes to improve rhizobia symbiotic performance under this challenging condition. We selected *Mesorhizobium*–chickpea symbiosis as a case study. This symbiotic interaction has been widely recognized for its high susceptibility to salt stress as previously reported in several studies [14,35,36,37], which makes it the perfect model to gain more insight into the mechanisms underlying the molecular and physiological responses of legume–rhizobium symbiosis to salinity.

## 2. Materials and Methods

### 2.1. Bacterial Strains: Origin and Growth Conditions

To evaluate the impact of salinity on *Mesorhizobium*–chickpea symbiosis, the *Mesorhizobium ciceri* strain LMS-1 was selected as a specific chickpea microsymbiont model [38]. To evaluate the effect of non-rhizobial nodule endophytes on the *Mesorhizobium*–chickpea symbiosis under salinity, four non-rhizobial bacterial endophytes, namely IRAMC:0104, IRAMC:0001, IRAMC:0142, and IRAMC:0077, were chosen for this study. Their selection was based on plant growth promotion screenings and compatibility tests with rhizobia conducted in our bacterial collection. These non-rhizobial endophytes were isolated as described by [39] from the root nodules of two native legumes, *Calobota saharae* and *Calicotome villosa*, grown in arid regions in South Tunisia. Molecular analysis of the 16S rRNA gene showed that IRAMC:0104, IRAMC:0001, IRAMC:0142, and IRAMC:0077 are identified as *Phyllobacterium salinisoli* (P1, NCBI accession number OR179644), *P. ifriqiyense* (P2, NCBI accession number OR179648), *Xanthomonas translucens* (X, NCBI accession number OR179647), and *Cupriavidus respiraculi* (C, NCBI accession number OR179643), respectively.

The yeast extract mannitol YEM (liquid and solid) medium [40] was chosen as a routine growth medium for all strains unless otherwise expressed.

### 2.2. Bacterial Salt Tolerance and Plant Growth Promoting Traits 

Bacterial tolerance to salinity was evaluated based on the capacity of each strain to grow on YEM solid medium supplemented with different concentrations of sodium chloride (170 mM, 340 mM, and 510 mM of NaCl) at 28 °C. Growth on normal YEM solid medium was used as a control. For each isolate, three plates were inoculated per treatment. Growth under stress (%) was calculated considering growth under control conditions (growth without NaCl) as 100% growth using the formula ((colony diameter under stress/colony diameter under control) × 100)).

For the in vitro screening of different plant growth promoting features, non-rhizobial endophytes were grown overnight in liquid medium YEM at 28 °C. After incubation, cells were collected and resuspended in sterile saline solution to an OD_600nm_ of 0.5 for each strain. All PGP activities were tested in triplicate for each non-rhizobial endophytic strain.

The solubilization of inorganic phosphate was evaluated using Pikovskaya medium plates [41], while the production of siderophores was studied using Chrome Azurol S (CAS) medium plates [42]. For both activities, 10 microliters of each bacterial culture were spotted onto the solid medium, and plates were incubated at 28 °C for 5–7 days. The appearance of a clearance zone around the colonies (a transparent zone for phosphate solubilization and an orange zone for siderophores production) was considered as positive for both PGP traits. The phosphate solubilization index (PSI) and the siderophores production index (SPI) were calculated using the formula ((halo diameter + colony diameter)/colony diameter) as previously described [43]. 

The 1-aminocyclopropane-1-carboxylate (ACC) deaminase activity of these endophytes was assessed under free-living conditions according to the methodology described by Brígido et al. [44]. Strains were grown in liquid YEM medium until reaching the stationary phase. Cells were washed, collected, and resuspended in N-free M9 medium supplemented with 5 mM of ACC to induce ACC deaminase activity in the bacterial cells. After incubation at 28 °C for 48 h, the enzymatic activity was determined based on the α-ketobutyrate produced as a result of the ACC cleavage by ACC deaminase, as described by Penrose and Glick [45]. Total protein content was quantified using the Bradford method [46]. *Pseudomonas putida* UW4 was used as positive control [47].

The ability of bacterial endophytes to produce IAA was estimated by the colorimetric method adopted by Brígido et al. [48]. IAA production was evaluated using individual or combined cell cultures under normal and two salt stress conditions. Briefly, 250 µL of each bacterial suspension with an initial OD_600nm_ of 0.5 were inoculated into tubes containing 4 mL liquid medium supplemented or not with tryptophan (250 µg/mL). After 48 h of incubation at 28 °C, bacterial cultures were centrifuged, and 1 mL of the supernatant was mixed with 2 mL of Salkowski reagent and incubated at room temperature for 20–30 min. The absorbance was measured at 535 nm. The concentration of IAA in each sample (individual strains or consortium) was determined using a standard curve of pure IAA (ranging from 0 to 100 µg/mL). To assess the potential impact of salt stress on bacterial ability to produce IAA, the growth was in liquid medium supplemented with 13 mM NaCl and 130 mM NaCl. 

### 2.3. Effect of Salt Stress on Biofilm Production

The capacity to produce biofilm under control and salt stress was performed using microtiter PVC plates as described in [49]. Briefly, free-living cells of the individual or the combined strains were inoculated in YEM without (YEM) or with salt (YEM + 13 mM NaCl, and YEM + 130 mM NaCl) and standardized to a final OD_600nm_ of 0.5. One hundred fifty microliters of each bacterial suspension were pipetted into individual wells of a 96-cell PVC plate. The plates were sealed with parafilm and incubated for 72 h at 28 °C. After incubation, cell growth was measured at 600 nm, and biofilm formation was quantified using the normalization of the absorbance of the remaining dye (OD_565nm_) by cell growth (OD_600nm_). A total of six replicates per treatment was performed under each condition.

### 2.4. Collection of Root Exudates and Analysis of Phenolic Compounds Composition

To evaluate the effect of salinity on the chickpea root exudates composition, the root exudates were collected from chickpea seedlings growing under normal conditions (NRE) or subjected to salt stress with 13 mM NaCl (SRE) for one week. The chickpea root exudates were collected as described by [50], using sterile distilled water instead of minimal medium. Briefly, chickpea seeds were initially surface sterilized and pre-germinated as described earlier [51]. The seedlings were transferred to separate 50 mL conical tubes with a sterile net placed slightly above 10 mL of sterile distilled water (normal conditions) or 13 mM NaCl solution (for salinity treatment). Aluminum foil was used to shield the tubes from light. Each tube contained a single seedling, and each treatment was replicated 10 times. The tubes were placed in a growth chamber for 7 days (one week) with a 16-h-light and 8-h-dark cycle and 24 °C day and 18 °C night temperature and 65% relative humidity as previously described [52].

After collection, root exudates were filter-sterilized and checked for contamination using Luria Bertani medium (incubated for 48 h at 28 °C). After confirming the absence of contamination, part of the root exudates was lyophilized for analysis of the composition of phenolic compounds and the other part was stored at −80 °C until further use in bioassays. The protein concentrations of root exudates were adjusted to a final concentration of 0.07 at 280 nm.

Phenolic compounds analysis was carried out on a 20 µL volume of exudates using a quadrupole mass spectrometer: LC-MS-2020 (Shimadzu, Kyoto, Japan) equipped with an electro-nebulization ionization source (ESI) in negative mode. The mass spectrometer was coupled in-line with an ultra-fast liquid chromatography system comprising an LC-20AD XR binary pump system, a SIL-20AC XR autosampler, a CTO-20AC column oven, and a DGU-20A 3R degasser (Shimadzu, Kyoto, Japan). An Inert sustain C18 column (GL Sciences Japan, Tsukuba, Japan) (150 mm × 3 mm, 3 μm) was applied for analysis. The mobile phase was composed of (0.02% acetic acid in H_2_O/ACN, (1/1, *v*/*v*) with linear isocratic elution: 10 min time of acquisition. The mobile phase flow rate was 0.4 mL/min, the column temperature was maintained at 40 °C, and the injection volume was 20 µL. Spectra were monitored in SIM (Selected Ion Monitoring) mode and processed using Shimadzu Lab solutions LC-MS version 5.42 software. High-purity nitrogen was used as a nebulizer and auxiliary gas. The phenolic compounds were identified by matching the obtained retention times and mass spectra to chemical standards of >98% purity obtained from Sigma Chemical Co. (St Louis, MO, USA).

### 2.5. Molecular Response of Mesorhizobium to Salt Stress and to Root Exudates Stimuli

To determinate the molecular response of LMS-1 to salt stress and to chickpea root exudates stimuli, total RNA was extracted from free-living cells exposed to salt stress (250 mM NaCl) and/or to chickpea root exudates collected under salt or normal conditions (SRE and NRE). 

For gene expression analysis, *Mesorhizobium ciceri* LMS-1 cells were grown in Tryptone Yeast (TY) liquid medium [53] at 28 °C. At the early exponential phase, the bacterial culture with an optical density of OD (600 nm) = 0.3 was subjected to different conditions by adding salt and/or root exudates, as follows: cells exposed to 250 mM NaCl (R + salt), cells exposed to NRE only (R + NRE), cells exposed to SRE only (R + SRE), and cells simultaneously exposed to 250 mM NaCl and to NRE (R + salt + NRE). All inocula were adjusted to same final volume (10 mL) using sterile distilled water, and three biological replicates per treatment were conducted. Cells were incubated for 6 h at 28 °C. After incubation, cell pellets were collected by centrifugation at 8000× *g* for five minutes and conserved at −80 °C for RNA extraction. 

The total RNA of the cells was extracted using the Direct-zolTM RNA MiniPrep plus Kit (Zymo Research, Freiburg, Germany) according to the manufacturer’s protocol, except for the lysis step, which consisted of the resuspension of cells pellet in 2 mL of lysis solution (1.4% sodium dodecyl sulfate, 4 mM EDTA, 75 μg of proteinase K) and incubated for 10 min at 65 °C. Following the extraction procedure, around 1 µg of total RNA under-went a DNA decontamination treatment using PerfeCta DNase I (Quanta BioSciences, Inc., Beverly, MA, USA). The conversion of total RNA to cDNA was accomplished using the NZY First-Strand cDNA Synthesis kit (NZYtech, Lisbon, Portugal). 

The evaluation of early symbiotic genes expression (*nodD* and *nodC*) and salt tolerance genes response (*exoY* and *otsA*) was performed by quantitative RT-PCR analyses (qRT-PCR). The selection of *exoY* and *otsA* genes was based on their previous reports as one of the most upregulated transcripts under salinity, along with their potential interfering in legume–rhizobium symbiosis [54,55]. All primers used herein are listed in Table 1.

The 16S rRNA gene was used as a reference gene for the normalization of the expression of the bacterial target genes [57]. The qPCR reactions were performed in a final volume of 16 µL, using 6 ng of cDNA, 8 µL of NZY qPCR Green Master Mix (2×) (NZYtech, Lisbon, Portugal), and 7.5 pmol of each primer. All qRT-PCR reactions were conducted in 96-well plate format and run on an Applied Biosystems™ 7500 Real-Time PCR (Thermo Fisher Scientific, Waltham, MA, USA) under the following amplification program: an initial denaturation for 10 min at 95 °C, 40 cycles of 95 °C for 15 s and 58 °C for 30 s. The specificity of amplification reactions was verified by a melting curve analysis performed at 58–95 °C. No template controls to detect DNA contamination or primer dimers were also performed. A set of 6 technical replicates was used for each experimental treatment with 2 technical replicates for each biological replicate. The calculation of normalized arbitrary units obtained by qPCR for the target genes was performed using the normalization factors obtained from the reference gene (16S rRNA). 

### 2.6. Effect of Salinity on Chickpea Seed Germination 

Chickpea seeds were surface sterilized as previously described [51]. After sterilization, the seeds were pre-germinated on 0.75% agar plates supplemented with NaCl at final concentrations of 0 mM, 13 mM, 17 mM, 34 mM, and 51 mM to test the effect of salt stress on seeds’ germination rate. 

### 2.7. Evaluation of Non-Rhizobia Nodule Endophytes Potential on Mesorhizobia-Chickpea Symbiosis under Salinity 

To evaluate the effect of non-rhizobial nodule endophytes from legumes native to arid environments on the *Mesorhizobium*–chickpea symbiosis under salinity, a plant growth assay was conducted in a plant growth chamber under controlled conditions. Pre-germinated seeds on 0.75% agar plates (without salt) were used for subsequent use in the plant trial. Seedlings (one seed per pot) were transferred to plastic pots filled with a sterile sand:vermiculite 1:2 (*v*/*v*) mixture. To test whether the co-inoculation with the non-rhizobial endophytic bacteria has an impact on legume–rhizobium symbiosis under salinity, the rhizobial and endophytic inocula were prepared as previously described [24]. One ml of inoculum containing rhizobial cells at an optical density of 600 nm (OD_600nm_) of 0.7 and an OD_600nm_ of 0.6 of each non-rhizobial endophytes was used to inoculate each chickpea seed as following: LMS-1 alone (R), LMS-1 with *Phyllobacterium salinisoli* and *Xanthomonas translucens* (R + P1 + X); LMS-1 with *P. salinisoli*, *P. ifriqiyense,* and *X. translucens* (R + P1 + P2 + X); LMS-1 with *P. ifriqiyense* and *Cupriavidus respiraculi* (R + P2 + C); and LMS-1 with *P. ifriqiyense*, *C. respiraculi,* and *X. translucens* (R + P2 + C + X). 

Plants were grown in a growth chamber under a 16-h-light and 8-h-dark cycle and 24 °C day and 18 °C night temperature with a relative humidity of 65% [52] and irrigated three times a week with a nitrogen-free-nutrient solution [58]. Uninoculated plants watered with a nutrient solution supplemented with nitrogen (0.1% KNO_3_) were used as positive controls (PC). Fifteen days after inoculation, salt stress was imposed through irrigation with -nutrient solutions supplemented with 13 mM NaCl and applied in alternate watering. Five biological replicates per treatment were used. Plants were harvested 5 weeks after sowing, and several parameters were measured, including shoot dry weight (SDW), root dry weight (RDW), number of nodules (NN), nodule fresh weight (NFW), and nodule dry weight (NDW). The Kjeldahl method was used for the estimation of total nitrogen content in plant shoots [59].

To evaluate the potential effects of non-rhizobial nodule endophytes co-inoculation on the internal morphology of chickpea nodules, nodule histology was performed on nodules from roots of 5-week-old plants as described earlier [49]. Nodules sections (2 µm) were examined by light microscopy under a Leica DM6000 B microscope (Leica, Wetzlar, Germany) and images were captured at different magnifications.

### 2.8. Statistical Analysis

Statistical analyses were carried out using SPSS 21.0 software (IBM Corp., Armonk, NY, USA). An independent samples *t*-test (*p* < 0.05) was performed to compare the means of phenolic compounds in chickpea root exudates. A heatmap of the phenolic compounds identified in the chickpea root exudates was generated using MetaboAnalyst 6.0 [60] after square root normalization of data.

Data obtained from the qRT-PCR experiment, chickpea seed gemination rate, bacterial growth, plant growth promoting features, biofilm production, and chickpea plant-growth assay were characterized by one-way analysis of variance (ANOVA) to compare the treatment or group means. The Duncan test was used to detect significant differences between the treatment or group means (*p* < 0.05). A two-way ANOVA was performed to evaluate the effect of salt stress and bacterial species on both IAA production and biofilm formation. 

## 3. Results

### 3.1. Bacterial Salt Tolerance and PGP Activities

In this study, all strains exhibited normal growth in media without salt. The *Meso-rhizobium* strain LMS-1 showed high sensitivity to salt stress, and its growth under salt stress was significantly lower compared to other non-rhizobial endophytes under same conditions. A 75% reduction of LMS-1 growth was found in the presence of 170 mM NaCl. In opposite, all non-rhizobial endophytic isolates were able to grow at gradually increased NaCl concentrations (up to 510 mM NaCl), except for *Cupriavidus respiraculi* IRAMC:0077 strain, which was more sensitive to concentrations above 170 mM NaCl (Table S1). 

In vitro screening of PGP traits showed that all non-rhizobial endophytic strains exhibited at least two PGP features (Table S2). All endophytes were positive for indoleacetic acid (IAA) production, although presenting different IAA production levels. *Phyllobacterium salinisoli* IRAMC:0104 and *Xanthomonas translucens* IRAMC:0142 were high IAA producers with a production ≥40 µg/mL, while the *Cupriavidus respiraculi* IRAMC:0077 strain was the lowest IAA producer. Similarly, all isolates were able to produce siderophores, but their production was low. The capacity of phosphate solubilization was only detected in *Phyllobacterium salinisoli* IRAMC:0104 with a high phosphate solubilization index. None of the tested endophytes showed ACC deaminase activity under free-living conditions (Table S2).

### 3.2. The Effect of Salt Stress on the Phenolic Compounds Composition of Chickpea Root Exudates 

As anticipated, exposure to salinity resulted in a significant alteration of the phenolic compounds profile of the chickpea root exudates. Chemical analysis revealed the presence of 13 phenolic compounds in root exudates collected under control conditions, while only 7 of these compounds were identified in those exudates exposed to salt stress (Table S3). Gallic acid, cholorogenic acid, 1,3-di-O-caffeoyquinic acid, rutin, and hyperoside (quercetin-3-o-galactoside) compounds were only detected in the root exudates collected under normal conditions, suggesting that salinity interferes with their production. Furthermore, the concentration of all mentioned compounds was significantly decreased in the root exudates collected under salinity conditions compared to those produced under normal conditions (*p* < 0.05) (Table S3 and Figure S1). These results demonstrate that chickpea exudation is affected by salinity, which may have a negative impact on the ability of both symbiotic partners to communicate and interact. 

### 3.3. Evaluation of Mesorhizobium Response to Salinity and Root Exudates Stimuli

To investigate the *Mesorhizbium* response to salt stress and/or to root exudates stimuli, gene expression analyses of symbiosis and salt tolerance related genes were performed by qRT-PCR. 

**Figure 1:**
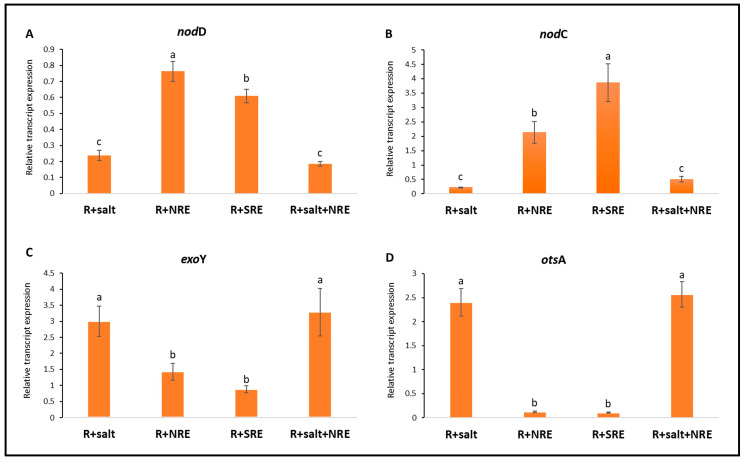
Transcription levels of early symbiotic genes and salt tolerance genes in LMS-1 cells exposed to salt stress and/or to chickpea root exudates. (**A**–**D**) correspond to the expression levels, determined by qRT-PCR, of *nodD, nodC*, *exoY*, and *otsA* genes, respectively. R + salt: LMS-1 exposed to salt (250 mM NaCl); R + NRE: LMS-1 exposed to normal root exudates; R + SRE: LMS-1 exposed to root exudates collected under salinity; R + salt + NRE: LMS-1 exposed to both salt (250 mM NaCl) and normal root exudates. Data are presented as the mean and standard error values of six independent technical replicates. Means followed by the same letter do not differ significantly by Duncan test (*p* > 0.05).

As expected, a significant upregulation (*p* < 0.05) of the *nodD* gene was observed in cells exposed to chickpea root exudates, either collected under normal conditions (NRE) or submitted to salinity (SRE) (Figure 1A). However, the response to the SRE was significantly lower than that obtained from the NRE, thus suggesting that changes in the composition of root exudates may have an impact on the expression of the *nodD* gene. A similar effect of root exudates was also registered in the *nodC* gene expression compared to cells unexposed to root exudates (Figure 1B). Interestingly, the level of *nodC* transcripts under the effect of SRE was significantly higher by about two times compared to exposure to NRE. In parallel, the induction of *nodC* and *nodD* genes in the absence of root exudates was residual. On the other hand, the expression of both symbiotic genes was inhibited in salt stressed cells exposed to NRE, suggesting that salinity *per se* has a greater impact on the *Mesorhizobium* response to root exudates stimuli than the changes on plant root exudation composition. 

A significant increase of the expression of *exoY* and *otsA* genes was detected in LMS-1 cells grown under salt stress (250 mM NaCl) either with or without exposure to root exudates (R + salt; R + S + NRE) compared to cells not exposed to salt stress (*p* < 0.05) (Figure 1C,D). Our data showed an increase of up to twofold in the expression of *exoY* gene in salt-stressed cells compared to those grown under exposition to root exudates (R + NRE; R + SRE). Similarly, a 10-fold augment of the level of *otsA* gene expression was registered within the two treatments R + salt and R + S + NRE. Furthermore, the *exoY* gene expression was induced by the presence of root exudates, while induction of *otsA* gene expression was residual (Figure 1C,D).

### 3.4. Chickpea Seed Germination and Effect of Non-Rhizobial Endophytes on Mesorhizobium Symbiotic Performance under Salinity

Chickpea seed germination was negatively affected by salinity, with a decrease in the germination percentage as the NaCl concentration increased. Notably, a significant reduction of 44.26% was obtained under 34 mM NaCl (Figure S2), while a decrease of 24.75% on germination was registered with the lowest level of salt stress (13 mM NaCl). Therefore, this concentration (13 mM NaCl) was selected to evaluate the effect of non-rhizobial endophytes isolated from nodules of legumes native to arid environments on the *Mesorhizobium*–chickpea symbiosis under salinity. 

**Figure 2:**
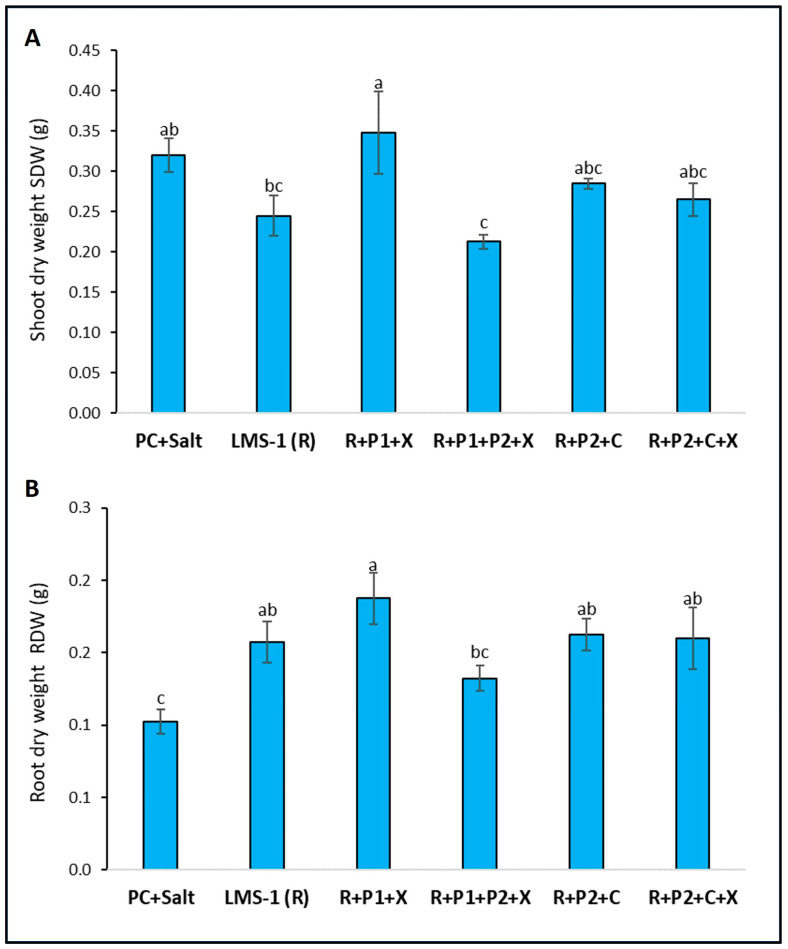
Effect of non-rhizobial endophytes co-inoculation on chickpea growth parameters under salinity: (**A**) shoot dry weight (SDW) and (**B**) root dry weight (RDW). PC + salt: uninoculated plants supplemented with synthetic nitrogen; LMS-1 (R): inoculated plants with LMS-1 alone; R + P1 + X: inoculated plants with LMS-1 + *Phyllobacterium salinisoli* + *Xanthomonas translucens*; R + P1 + P2 + X: inoculated plants with LMS-1 + *P. salinisoli + P. ifriqiyense* + *X. translucens*; R + P2 + C: inoculated plants with LMS-1 + *P. ifriqiyense* + *Cupriavidus respiraculi*; R + P2 + C + X: inoculated plants with LMS-1 + *P. ifriqiyense* + *C. respiraculi* + *X. translucens*. Error bars represent the standard error. Different letters correspond to statistically significant differences by Duncan test (*p* < 0.05).

In terms of plant growth parameters, only the consortium containing the non-rhizobial endophytes *Phyllobacterium salinisoli* IRAMC:0104 and *Xanthomonas translucens* IRAMC:0142 (R + P1 + X) strains resulted in significant promotion of the shoot dry weight compared to plants single inoculated with *Mesorhizobium ciceri* LMS-1 (R) (Figure 2A). The shoot dry weight of plants co-inoculated with this consortium showed an increase of 42.04% compared to LMS-1 alone and 12% compared to the positive control plants. All treatments of inoculated plants resulted in significant promotion of root dry weight compared to non-inoculated plants (positive control) apart from treatment R + P1 + P2 + X.

**Figure 3:**
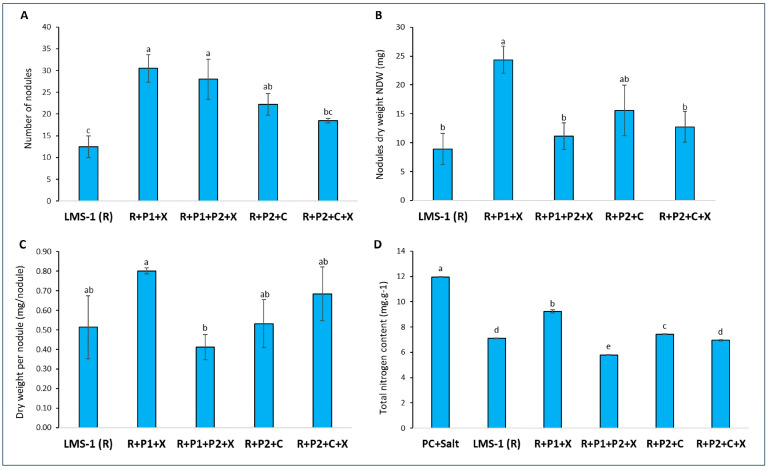
Effect of co-inoculation with non-rhizobial bacterial endophytes isolated from legumes grown on arid environments on *Mesorhizobium* symbiotic performance under salt stress: (**A**) number of nodules, (**B**) nodule dry weight (NDW), (**C**) average weight per nodule, and (**D**) total nitrogen content. PC + salt: uninoculated plants supplemented with nitrogen; LMS-1 (R): inoculated plants with LMS-1 alone; R + P1 + X: inoculated plants with LMS-1 + *P. salinisoli* + *X. translucens*; R + P1 + P2 + X: inoculated plants with LMS-1 + *P. salinisoli* + *P. ifriqiyense* + *X. translucens*; R + P2 + C: inoculated plants with LMS-1 + *P. ifriqiyense* + *C. respiraculi*; R + P2 + C + X: inoculated plants with LMS-1 + *P. ifriqiyense* + *C. respiraculi* + *X. translucens*. Data correspond to the mean and standard error values of 5 biological replicates. Different letters correspond to statistically significant differences by Duncan test (*p* < 0.05).

In terms of the symbiotic performance of *M. ciceri* LMS-1, a trend towards an increase in the number of nodules formed by the *Mesorhizobium* symbiont was observed when co-inoculated with different consortia containing non-rhizobial endophytic bacteria under salinity (Figure 3A). In fact, co-inoculation of *M. ciceri* LMS-1 with the two endophytic strains *P. salinisoli* and *X. translucens* (R + P1 + X) significantly increased chickpea nodulation by more than 2.5 times. Similarly, the nodule fresh weight (NFW) and nodule dry weight (NDW) in plants inoculated with this consortium were significantly enhanced by 150% and 173.59%, respectively, when compared to those obtained in plants inoculated with *M. ciceri* LMS-1 alone (Figure 3B and Figure S3). 

Despite that the average weight per nodule greatly varied among the treatments, no significant differences were observed between the average weight per nodule in co-inoculated plants and those single inoculated. Nevertheless, co-inoculation with the consortium R + P1 + X resulted in an increase of 56.82% in the average weight per nodule compared to the ones formed in single-inoculated plants. Nodules formed by this consortium (R + P1 + X) were significantly bigger than nodules of plants inoculated with *P. salinisoli*, *P. ifriqiyense,* and *X. translucens* (R + P1 + P2 + X), which presented the smallest nodules among all treatments (Figure 3C). Moreover, this improvement in the symbiotic behavior of rhizobia in the presence of non-rhizobial endophytes was also reflected through the level of nitrogen content in plant shoots. The combined inoculation with R + P1 + X induced a significant increase of about 30.14% in total nitrogen content in comparison to inoculation with LMS-1 alone (Figure 3D).

### 3.5. Analysis of Nodule Histology

**Figure 4:**
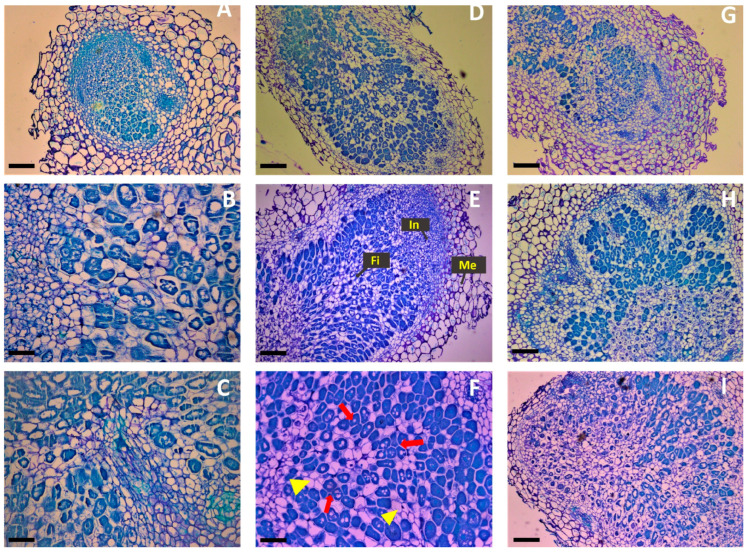
Effect of co-inoculation with endophytes from an arid environment on rhizobia nodules development under salt stress. Bright field micrographs of nodules histological sections. (**A**–**C**) Nodules from rhizobia alone, (**D**–**F**) nodules from R + P1 + X, (**G**) nodules from R + P1 + P2 + X, (**H**) nodules from R + P2 + C, and (**I**) nodules from R + P2 + C+X. Me: meristematic zone, In: infection zone, and Fi: fixation zone. Magnification in (**A**,**D**,**E**,**G**–**I**) is 40×; (**B**,**C**,**F**) is 63×. The red arrows in (**F**) indicate differentiated bacteroids, and the yellow arrowheads indicate uninfected cells.

To assess whether the presence of non-rhizobial endophytes induced changes in the development of nodules formed by *Mesorhizobium*, histological sections of root nodules from all treatments were made and their morphology was compared with that of root nodules from plants inoculated only with *Mesorhizobium*. All nodules exhibited a normal histology of effective nitrogen-fixing nodules, which includes a well-defined meristematic zone along with infection and nitrogen fixation zones (Figure 4A,D,E,G–I). The senescence zone was not observed in any of the analyzed nodules. Besides the difference in size of the nodules formed in the treatments R + P1 + X and R + P1 + P2 + X, nodules from the treatment R + P1 + X showed the biggest fixation zone with a higher number of well-differentiated bacteroids compared to nodules formed solely by rhizobia (Figure 4D–F). On the other hand, nodules of plants inoculated with R + P1 + P2 + X consortium contained a higher proportion of undifferentiated bacteria, namely immature bacteroids (Figure 4G). Additionally, no significant changes in the phenotype of infection threads and release pockets were observed between the nodules of the different treatments. In most nodules, the bacteroid organization within the cortical cells was well defined and similar among treatments (Figure 4). Altogether, these results suggest that the consortium R + P1 + X facilitated the *Mesorhizobium* infection ability and nodule development under salt stress.

### 3.6. Effect of Salt Stress on IAA Production and Biofilm Formation

To investigate whether the positive effects of the consortium R + P1 + X on chickpea growth and symbiosis were due to synergistic effects of the bacteria on IAA production or biofilm formation, the capacity of the microsymbiont *M. ciceri* LMS-1 and the two endophytic strains *P. salinisoli* and *X. translucens* to produce IAA and to form biofilm was tested individually or in consortium under normal and salt stress (13 mM NaCl and 130 mM NaCl ) conditions.

As expected, the exposure to salt stress negatively influenced the capacity of all three individual strains to produce IAA under free-living conditions (F = 4434, *p* < 0.001). A shift in the levels of IAA production was observed in response to salinity compared to control conditions (Table 2 and Table S2). Furthermore, our results showed that IAA production levels were influenced by bacterial species (F = 2185, *p* < 0.001), where variable amounts of IAA were produced among the strains tested. For example, the ability of the non-rhizobial bacterial endophyte *P. salinisoli* to synthesize high levels of IAA was not as affected by salinity (130 mM of NaCl) as in *Mesorhizobium* and *Xanthomonas* (Table 2). 

Contrary to our expectations, the combination of *Mesorhizobium* and the two endophytic strains had no positive influence on IAA production (Table 3). On the contrary, the amounts of IAA produced by the bacterial consortium were much lower than those produced by any of the strains individually, especially under salt stress conditions (Table 2 and Table 3). 

Contrary to IAA production, the LMS-1 strain was the one that exhibited the highest levels of biofilm production, while the two non-rhizobial strains (*P. salinisoli* and *X. translucens*) presented negligible amounts of biofilm under both control and salinity conditions (Table 2 and Table S2). More interestingly, the exposure to salt stress substantially increased biofilm development by *Mesorhizobium*, which is consistent with results from genes expression, where a significant increase in the expression level of *exoY* gene was observed in salt-stressed cells (Table 2). This result confirms that biofilm production is one of the osmoadaptative responses adopted by the LMS-1 strain against salinity. Our results showed that biofilm formation was influenced by both the bacterial species (F = 436.6, *p* < 0.001) and the salinity levels (F = 7.13, *p* < 0.05). 

As observed in IAA production, the biofilm production was not significantly improved with the combination of LMS-1 with the two endophytic strains (R + P1 + X). In fact, very low amounts of biofilm were produced by this consortium under normal and salt stress conditions compared to those produced by *Mesorhizobium* alone (Table 3). 

## 4. Discussion

Most legumes demonstrate high sensitivity to salinity, and chickpea is one of the most sensitive legumes. Several studies have confirmed the harmful effects of salinity on the germination and growth of chickpea [14,24,61]. Similarly, in this study, the germination rate of chickpea seeds decreases with the increasement of NaCl concentrations, confirming the susceptibility of this legume to salinity. On the other side, the *Mesorhizobium* strain is less sensitive to salt stress than its host, with its growth significantly reduced under 250 mM NaCl. This result agrees with the fact that, in general, *Mesorhizobium* strains are more sensitive to salt stress than *Rhizobium* and *Sinorhizobium* strains but are more tolerant to salt stress than *Bradyrhizobium* strains [62,63]. 

Although the salt tolerance of each symbiotic partner was different, it is known that even minor soil salinity levels have the potential to disrupt the symbiotic balance between plants and their microbial partners [64]. The initiation of the symbiotic legume–rhizobium interaction relies on the exchange of chemical signals between both partners, which starts with legumes releasing a cocktail of flavonoids through root exudates. In turn, rhizobial perception of these compounds triggers the synthesis and secretion of Nod factors encoded by *nod* genes [8]. However, salinity, as other abiotic stresses, can alter the composition of root exudates, which may affect the early steps of the legume–rhizobium symbiosis. In fact, a decrease in the capacity of soybean root exudates collected under salt stress to induce *nod* genes expression was reported by Dardanelli et al. [65]. Herein, salinity led to a significant change in the composition of phenolic compounds of chickpea root exudates, which is in agreement with a recent study [66]. Moreover, these changes also differently affected the *nod* genes expression in *Mesorhizobium ciceri* LMS-1 strain. For instance, although expression of the *nodD* gene in LMS-1 was activated in response to both types of root exudates, i.e., collected under normal and salinity conditions, its expression was significantly lower in cells exposed to root exudates collected under salinity conditions. Our results corroborate that the activation of *nodD* gene in *Mesorhizobium ciceri* is highly dependent on the combination and composition of inducers and anti-inducers in chickpea root exudates [67]. In opposite, the expression of *nodC* gene was significantly higher when the LMS-1 strain was exposed to root exudates collected under salinity compared to normal conditions. Similar results were previously reported for a native peanut rhizobial strain, where an increase in *nodC* gene induction was detected in cells exposed to root exudates collected under acid stress [68]. Prior to that, the expression of nodulation genes in *R. meliloti* was also induced using *Medicago* root exudates collected at pH 5.8 [69]. In addition, a remarkable decrease in the expression of *nod* (both *nodD* and *nodC*) genes was observed in salt-stressed cells exposed to root exudates collected under normal conditions. This result indicates that, under such stress conditions, *Mesorhizobium* is unable to respond to the root exudates stimuli. Our results agree with Dardanelli et al. [70], who reported also a lower induction of *nod* genes in salt-stressed *Rhizobium etli* ISP42 cells exposed to bean root exudates compared to unstressed cells.

Besides the important role of exopolysaccharides (EPS) biosynthesis in rhizobium–legume symbiosis, namely in the infection process and bacteroid and nodule development [64], EPS production is also one of the osmoadaptative responses adopted by rhizobial strains to bypass salt stress [71]. Another strategy in bacteria includes the accumulation of certain compatible solutes, such as trehalose, to maintain an osmotic balance with their surrounding environment. Trehalose biosynthesis, encoded by *otsAB* genes in *Mesorhizobia*, is involved in abiotic stress responses and rhizobial symbiotic interactions [55]. Several studies have demonstrated that a high concentration of trehalose in bacteroids at the onset of nitrogen fixation protects cells and proteins from environmental stresses [72]. As expected, the expression of the *exoY* and *otsA* genes of LMS-1 were highly induced in LMS-1 cells exposed to salinity. Similarly, high induction of the *exoY* and *exoN* genes, which are responsible for EPS biosynthesis, was registered in *Sinorhizobium meliloti* strain 1021 when affected by salt stress [73]. The involvement of each of these genes in different stages of legume–rhizobium symbiosis explains the differences in expression upon root exudates stimuli in the absence of salt stress. Interestingly, the induction of *exoY* gene in LMS-1 cells exposed to salinity was twofold higher than when exposed to root exudates stimuli, suggesting that salt stress rapidly triggers the EPS biosynthesis in the LMS-1 strain. 

The effect of non-rhizobial endophytic bacteria, isolated from nodules of legumes native to arid regions, on the symbiotic performance of the mesorhizobial LMS-1 strain and the tolerance of chickpea to salinity conditions was evaluated in this study. Our results showed a notable enhancement in the overall growth of chickpea plants when co-inoculated with one of the non-rhizobial endophytic consortia tested (R + P1 + X). The combination of LMS-1 and the two endophytes *Phyllobacterium salinisoli* and *Xanthomonas translucens* resulted in a beneficial interaction, which was reflected through the amelioration of salinity in chickpea growth parameters. In agreement with these findings, previous studies also highlighted the positive effects of chickpea co-inoculation with *Mesorhziobim* and some endophytic strains under abiotic stress, such as manganese toxicity [24], salinity [24,49,74,75], and drought [76]. However, not all combinations of non-rhizobial endophytic bacteria tested here resulted in benefit to chickpea, which is consistent with previous studies [52,77]. 

Moreover, our results show that the increment of overall chickpea biomass in the R + P1 + X treatment may be a result of benefits in both the nodulation and nitrogen fixation efficiency of LMS-1. Earlier studies have also shown that the use of consortia containing non-rhizobial endophytic bacteria benefits the symbiotic performance of nitrogen-fixing symbionts, both nodulation and nitrogen fixation efficiency [24,49,52,78]. Here, only the treatment R + P1 + X presented a significant increase in overall symbiotic parameters (i.e., number of nodules, nodule dry weight, etc.), leading to an enhancement of both nodules’ formation and biological nitrogen fixation in chickpea. This improved behavior is likely the result of the beneficial functions provided by the endophytic isolates to the plant itself, which may have facilitated the legume-rhizobium interaction under salinity conditions. Plants growing under salinity suffer the negative impacts of oxidative stress due to salt ions accumulation. The increase of reactive oxygen species (ROS) causes growth inhibition, cell membrane damage, and DNA and photosynthetic pigments degradation [79]. However, various studies reported that the application of plant growth promoting bacteria can mitigate these adverse impacts either by the induction of plant genes coding for the synthesis of ROS scavengers or by the production of bacterial antioxidative enzymes [80,81]. Taking into account their capacity to tolerate increased salt concentrations (up to 510 mM NaCl), it is possible that co-inoculation with *P. salinisoli and X. translucens* have induced the antioxidant defense system of chickpea plants. In fact, plants co-inoculated with the consortium R + P1 + X showed the highest shoot development, thus overcoming not only the effect of rhizobium alone but also the effect of synthetic nitrogen. This improvement in chickpea growth indicates stress relief in plant shoots, which can have a direct influence on *Mesorhizobium*–chickpea symbiotic interaction. 

In addition, phosphate solubilization, IAA, and siderophores production may also account for the improvement of legume–rhizobium interactions and plant tolerance to abiotic stress [82,83,84,85]. Plant hormones are known to play an essential role in plant–microorganism interactions, including in the regulation of symbiotic legume–rhizobium interactions and root nodule organogenesis [86]. It may be possible that the presence of endophytic bacteria has contributed to a phytohormone balance (mainly auxins and cytokinin), promoting greater development of the root nodules thus leading to a larger number of nitrogen-fixing bacteroids within the nodules. Consistent with this, the histological analysis of nodules sections revealed that the nodules induced in co-inoculated plants with R + P1 + X presented a bigger fixation zone with a higher number of differentiated bacteroids compared to nodules induced by rhizobia alone. While the two endophytes *P. salinisoli and X. translucens* showed good levels of IAA production under salinity conditions, the levels of IAA produced by the bacterial consortium R + P1 + X were much lower than those of individual strains under free-living conditions, thus suggesting that this mechanism may not be involved in promoting the *Mesorhizobium*–chickpea symbiosis. Notwithstanding, it is important to highlight that these results were obtained without accounting for the role of the plant in modeling microbe–microbe interactions. In fact, several studies have demonstrated the crucial role of plants in shaping the rhizosphere microbiome. For instance, Liu et al. [87] reported the effect of soybean genotype on the alteration of rhizosphere microbiome assembly. In addition, recent studies show that several plant metabolites affect the interaction of plant with microbes below ground [88] and vice versa [89]. Moreover, plants exposed to abiotic stress, including salinity, launch a ‘cry for help’ that is transmitted through root exudation. Released into the rhizosphere, these signaling molecules help recruit selective groups of microbes to colonize their root system and subsequently contribute to the improvement of plant health [90]. In this regard, we can speculate that the in vivo interactions between *Mesorhizobium* and the two endophytic bacterial strains were shifted by the host plant in a way that their result contributes positively to plant health, which directly and/or indirectly benefits the nodulation and nitrogen fixation processes. 

To this end, further studies are needed to better understand the mechanisms of colonization adopted by these endophytes in different plant organs (roots and nodules) and how plant–endophyte interactions modulate nodule organogenesis and development. These results highlight the beneficial effects of co-inoculation with non-rhizobial endophytes from legumes native to arid environment and reveal their potential as a third partner in the establishment and development of legume–rhizobium symbiosis under salinity conditions.

## 5. Conclusions

Our study demonstrates that salinity affects the phenolic compounds composition of chickpea root exudates, and these changes have implications on the *Mesorhizobium ciceri* LMS-1 response during the early stages of the symbiotic legume–rhizobium interaction. Moreover, our study demonstrated that two non-rhizobial endophytes, *Phyllobacterium salinisoli* and *Xanthomonas translucens*, were able to improve the *Mesorhizobium* symbiotic performance and the chickpea growth under salinity. Therefore, these endophytes may be potential candidates to improve the productivity of one of the most salt-sensitive and most produced grain legumes worldwide.

## Figures and Tables

**Table 1:** Target genes, reference gene, and primers used in qPCR.

Gene	Primers (5′—3′)	Target Size	Reference
*nodD*	F: TCCGGCACAGCTCGTATAG R: TTGGAGGGTCTCGGTGAATG	120 bp	This study
*nodC*	F: ATCCCGGTACATCACGCCTA R: GCTGAGCACGAAATCTCCAG	126 bp	This study
*exoY*	F: GCACATCCGCCGTCTACTAT R: TGATGATGATGCGAACGTCC	156 bp	This study
*otsA*	F: GATCATGGTGGCCGAACATC R: GACGAATTCCTTTGCGACGA	118 bp	This study
16S rRNA	IntF: GCTYAACSTGGGAACTGC IntR: TTTACRGCGTGGACTACC	199 bp	[56]

**Table 2:** Effect of salt stress on IAA and biofilm production for individual strains.

Strains	IAA Production		Biofilm Formation	
	13 mM NaCl	130 mM NaCl	13 mM NaCl	130 mM NaCl
*M. ciceri* LMS-1	8.16 ±0.67 ^c^	1.73 ±0.22 ^b^	7.77 ±1.09 ^a^	7.01 ±1.08 ^a^
*P. salinisoli*	27.48 ±0.6 ^a^	22.5 ±0.21 ^a^	0.63 ±0.08 ^b^	0.5 ±0.06 ^b^
*X. translucens*	13.92 ±0.41 ^b^	0.63 ±0.18 ^c^	1.3 ±0.23 ^b^	0.51 ±0.1 ^b^

Data represent the means and standard deviation of three (IAA) or six (biofilm) independent replicates. Significant differences are indicated with different letters in the same column by Duncan test (*p* < 0.05).

**Table 3:** Bacteria–bacteria interaction: IAA and biofilm production by the consortium (R + P1 + X).

Condition	Features	
	IAA Production	Biofilm Formation
Control	7.87 ±0.75 ^a^	0.72 ±0.12 ^b^
13 mM NaCl	5.87 ±0.98 ^b^	0.62 ±0.12 ^b^
130 mM NaCl	0.51 ±0.14 ^c^	0.95 ±0.07 ^a^

Data represent the means and standard deviation of three (IAA) or six (biofilm) independent replicates. Significant differences are indicated with different letters in the same column by Duncan test (*p* < 0.05).

## Data Availability

All the required data are available in the manuscript.

## Supplementary Materials

The following supporting information can be downloaded at: https://www.mdpi.com/article/10.3390/biology13020096/s1.
