# Supplementary material for: Bacterial Endophytes from Legumes Native to Arid Environments Are Promising Tools to Improve Mesorhizobium–Chickpea Symbiosis under Salinity
Source: Biology (Basel). 2024 Feb 3;13(2):96. doi: 10.3390/biology13020096 (PMC10886315; doi:10.3390/biology13020096)
Supplement: Supplementary file 1 [file biology-13-00096-s001.zip › biology-2805979-supplementary.pdf]

Supplementary information

Table S1. Bacterial growth under control and salt stress.

Bacterial strains	Growth without NaCl (%)	Growth with NaCl (%)		
		170 mM NaCl	340 mM NaCl	510 Mm NaCl
LMS-1	100	24.36 ±2.6 [b]	-	-
P. salinisoli	100	99.16 ±0.9 [a]	98.95 ±1.8 [a]	76.66 ±2.8 [a]
P. ifriqiyense	100	98.71 ±2.2 [a]	97.43 ±3.6 [a]	74.35 ±4.4 [a]
X. translucens	100	99.93 ±1.5 [a]	95.09 ±4.4 [a]	77.08 ±3.8 [a]
C. respiraculi	100	97.61 ±2.0 [a]	83.33 ±4.1 [b]	22.61 ±2.0 [b]

(-) No growth; Growth (%) was calculated considering growth under control conditions (without NaCl) as 100% growth for each bacterial species. Data represent the means and standard deviation of three independent replicates. Different letters in the same column indicate significant differences by Duncan test ($p < 0.05$).

Table S2. Phenotypic characterization of bacterial strains: plant growth-promoting features and biofilm formation.

Bacterial strains	IAA production	Index of phosphate solubilization (PSI)	Biofilm production	Index of siderophores production (SPI)	ACC deaminase activity
LMS-1	18.88 ±2.2[c]	nd	4.99 ±0.38 [a]	nd	- [51]
P. salinisoli	42.5 ±2.76 [a]	2.65 ±0.15 [a]	0.53 ±0.08 [c]	1.44 ±0.05 [a]	-
P. ifriqiyense	31.76 ±0.75 [b]	-	nd	1.3 ±0.05 [ab]	-
X. translucens	39.68 ±1.78 [a]	-	1.27 ±0.26 [b]	1.22 ± 0.11 [c]	-
C. respiraculi	16.3 ±1.17 [c]	-	nd	1.46 ±0.05 [a]	-

(-) No activity; (nd) Not determined; Data represent the means and standard deviation of three independent replicates. Significant differences are indicated with different letters in the same column by Duncan test ($p < 0.05$).

Table S3. Phenolic compounds composition of *Cicer arietinum* root exudates collected under control and salt stress conditions.

**Compound**	**Control** (ppm/mg)	**Salinity** (ppm/mg)
Quinic acid	10.331 ±0.469 [a]	0.037 ±0.005 [b]
Gallic acid	0.064 ±0.016 [a]	-
Protocachuic acid	0.399 ±0.016 [a]	0.121 ±0.003 [b]
Cholorogenic acid	0.035 ±0.002 [a]	-
Caffeic acid	6.906 ±0.055 [a]	0.044 ±0.003 [b]
1,3-di-O-caffeoyquinic acid	33.871 ±2.716 [a]	-
Syringic acid	3.983 ±0.166 [a]	0.729 ±0.026 [b]
Epicatechin	1.753 ±0.189 [a]	0.016 ±0.004 [b]
Rutin	0.177 ±0.007 [a]	-
Hyperoside (quercetin-3-o-galactoside)	0.527 ±0.008 [a]	-
Quercetrin (quercetin-3-o-rhamonoside)	0.872 ±0.008 [a]	0.050 ±0.005 [b]
Apegenin-7-o-glucoside	0.301 ±0.011[a]	0.008 ±0.001 [b]
Salviolinic acid	0.586 ±0.014 [a]	-

(-) Absence of the compound; Data represent the means and the standard deviation of three independent replicates under each condition; Different letters indicate statistical significance in the rows by T-test ($p < 0.05$).

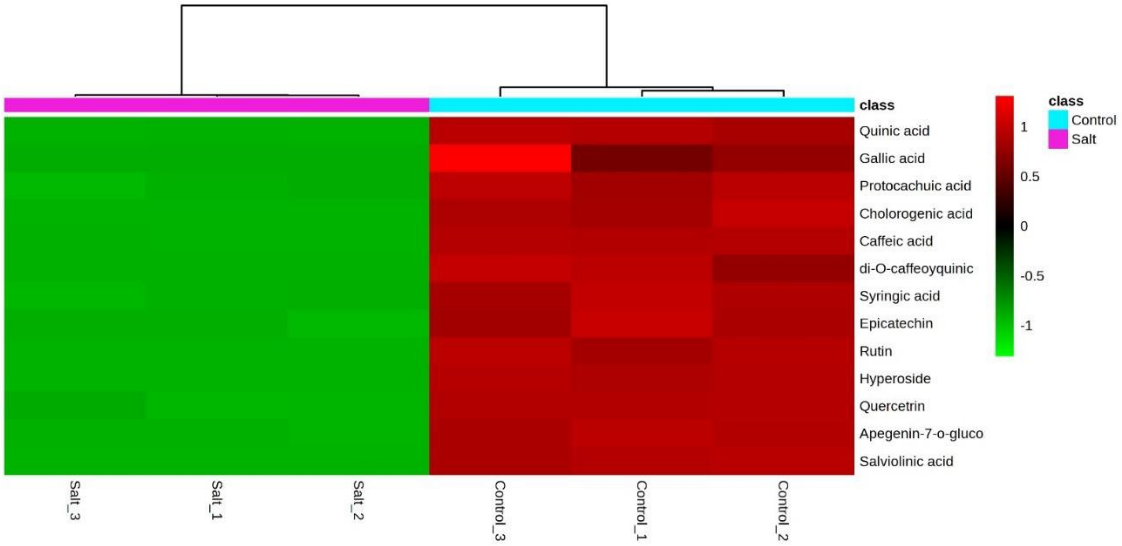

Figure S1. Heat map representing abundance of the phenolic compounds in the chickpea root exudates exposed to normal and salt stress conditions. Results from three independent biological replicates for each treatment are shown. Control_ denotes for root exudates collected under normal conditions and Salt_ denotes for root exudates collected under salt stress. 1, 2 and 3 represent the biological replicate number within each condition/treatment. The color scale ranges from: light green color corresponding to the absence or minimal abundance of the phenolic compound to red color corresponding to maximum abundance of phenolic compound.

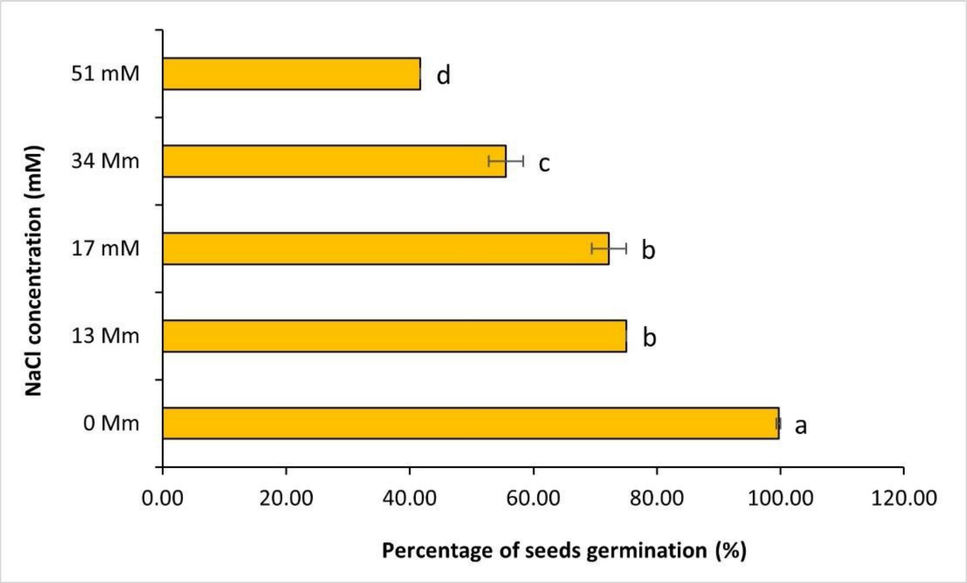

Figure S2. Effect of salt stress on seeds germination of chickpea. Data represent means and standard error of three independent replicates of 20 seeds per replicate. Different letters indicate statistical significance by Duncan test ($p < 0.05$).

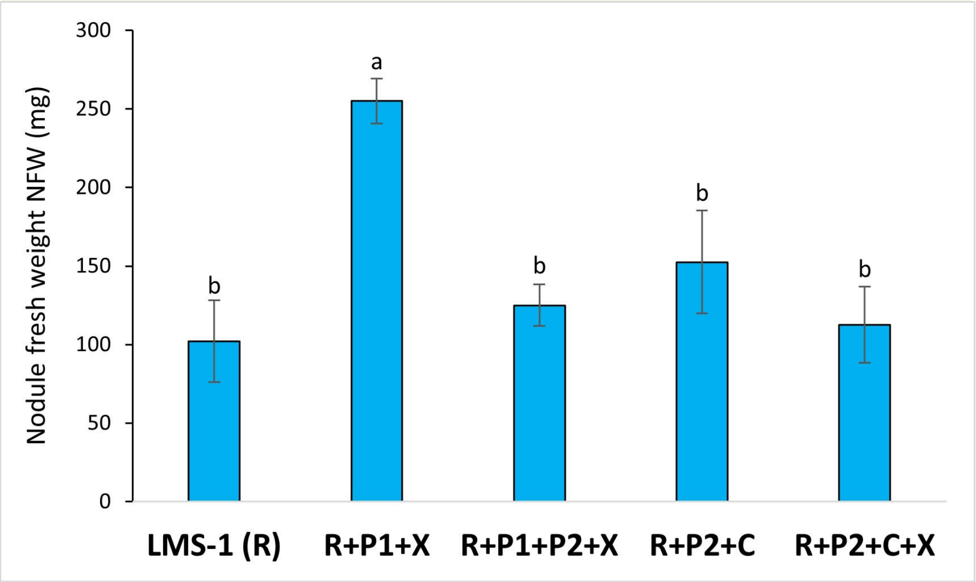

Figure S3. Effect Effect of co-inoculation with non-rhizobial endophytes from arid environment on *Mesorhizobium* nodule fresh weight (NDW) under salt stress. LMS-1 (R): inoculated plants with LMS-1 alone; R+P1+X: inoculated plants with LMS-1+*P. salinisoli* + *X. translucens*; R+P1+P2+X: inoculated plants with LMS-1 + *P. salinisoli* + *P. ifriqiyense* + *X. translucens*; R+P2+C: inoculated plants with LMS-1 + *P. ifriqiyense* + *C. respiraculi*; R+P2+C+X: inoculated plants with LMS-1 + *P. ifriqiyense* + *C. respiraculi* + *X. translucens*. Error bars represent the standard error. Different letters indicate statistical significance by Duncan test ($p < 0.05$).
